# Hyperbaric Oxygen Therapy as a Renewed Hope for Ischemic Craniomaxillofacial Diseases

**DOI:** 10.3390/healthcare13020137

**Published:** 2025-01-13

**Authors:** Chan He, Dou Huang, Lei Liu

**Affiliations:** 1 State Key Laboratory of Oral Diseases, West China Hospital of Stomatology, Sichuan University, Chengdu 610041, China; 2022224035198@stu.scu.edu.cn (C.H.); huangdou@zju.edu.cn (D.H.); 2 National Center for Stomatology, West China Hospital of Stomatology, Sichuan University, Chengdu 610041, China; 3 National Clinical Research Center for Oral Diseases, West China Hospital of Stomatology, Sichuan University, Chengdu 610041, China; 4 Department of Traumatic and Plastic Surgery, West China Hospital of Stomatology, Sichuan University, Chengdu 610041, China

**Keywords:** hyperbaric oxygen therapy, ischemic craniomaxillofacial diseases, medication-related osteonecrosis of the jaw, osteoradionecrosis, chronic jaw osteomyelitis, wound healing

## Abstract

Although the advancements in craniomaxillofacial surgery have been significant, ischemic craniomaxillofacial diseases remain challenging to treat due to insufficient blood supply. Hyperbaric oxygen therapy (HBOT) has emerged as a promising adjunctive treatment, exhibiting the potential to promote angiogenesis, exert anti-inflammatory effects, enhance bone regeneration, and possess antibacterial properties. Numerous studies have demonstrated its efficacy in stimulating healing processes, particularly in cases such as medication-related osteonecrosis of the jaw, osteoradionecrosis, chronic jaw osteomyelitis, and refractory wounds. Hyperbaric oxygen therapy not only accelerates healing and shortens recovery times but also reduces postoperative complications, infection risks, and enhances patients’ overall quality of life. This review aims to synthesize the research progress on the application of hyperbaric oxygen therapy in ischemic craniomaxillofacial diseases, providing a valuable reference for clinicians.

## 1. Introduction

Refractory ischemic craniomaxillofacial diseases refer to conditions caused by trauma, infection, radiation, or medication that result in damage to the blood supply in craniomaxillofacial tissues, leading to chronic local tissue necrosis and infection. The common refractory ischemic diseases include medication-related osteonecrosis of the jaw, tissue damage or necrosis following radiotherapy, chronic osteomyelitis of the jaw, and hard-to-heal wounds. The commonality among these diseases lies in the critical role of ischemia, hypoxia, and low tissue vitality resulting from local blood supply damage, which are also the main challenges in treatment. Traditionally, the primary treatment for these diseases has been surgical intervention. However, surgery can only remove necrotic tissue and is ineffective for areas with low tissue vitality. With the widespread application of hyperbaric oxygen therapy (HBOT), numerous studies have confirmed that it can enhance local oxygen levels and promote angiogenesis and anti-inflammatory effects, addressing the issues of hypoxia, low blood flow, and low vitality in these conditions [1,2]. This indicates that HBOT has significant adjunctive therapeutic value for treating refractory ischemic craniofacial diseases, providing new directions for patient recovery.

Refractory ischemic craniomaxillofacial diseases often prove difficult to cure or manage, characterized by prolonged disease courses and accompanied by severe and persistent symptoms. Insufficient blood supply to tissues is the main reason for the difficulty in healing these diseases. As the blood supply diminishes, the oxygen delivery to tissues becomes insufficient, resulting in a state of prolonged oxygen deprivation. This sustained lack of oxygen can exacerbate tissue ischemia and induce inflammation and necrosis, ultimately leading to a vicious cycle of further tissue damage and functional impairment. Such conditions significantly impact patients’ quality of life and also impose a heavy burden on families and society.

HBOT has garnered increasing attention in the medical field in recent years as an emergent treatment modality. By creating a hyperoxic environment, HBOT stimulates the cellular repair mechanisms and enhances tissue viability [3]. In the management of refractory ischemic craniomaxillofacial diseases, the traditional surgical interventions typically only address completely necrotic lesioned tissues and often fail to achieve a complete cure. Even when a cure is attained, recurrences are frequent. For example, patients with medication-related osteomyelitis of the jaw frequently encounter wound non-healing and recurrent infections accompanied by pus discharge, bone exposure, and persistent pain following surgery. HBOT, by boosting the oxygen supply to craniomaxillofacial tissues and fostering angiogenesis and cellular regeneration, presents a novel therapeutic option for these patients and demonstrates promising potential for application.

Currently, numerous scholars have conducted extensive and in-depth research on the mechanisms and effects of HBOT [4,5]. Many studies have indicated that HBOT’s ability to facilitate healing in damaged tissues is attributed to its anti-inflammatory and antimicrobial properties, as well as its role in promoting osteogenesis and angiogenesis [6,7,8]. Additionally, HBOT stimulates the secretion of various growth factors, thereby accelerating tissue repair and healing processes [9]. Furthermore, based on the continually expanding fundamental research findings, there is a growing consensus among researchers that HBOT can modulate immune system function and contribute to the repair of nerve damage [10,11,12].

Although many studies [13,14,15] have shown that HBOT offers a new avenue for the treatment of refractory ischemic craniomaxillofacial diseases, to date, no review has been published on this topic. Moreover, many clinicians remain unaware of its potential applications. In light of this, combined with our own clinical experience, we conducted a literature review and provide an overview of this research area for the first time. This review aims to provide an overview of the current application of HBOT in refractory ischemic craniomaxillofacial diseases by analyzing its mechanisms, effects, and considerations in clinical practice, serving as a reference for future research and practice.

## 2. Materials and Methods

### 2.1. Search Strategy

The comprehensive literature review was conducted using multiple databases: Cochrane, Embase, PubMed, and Web of Science. Mesh terms such as ‘hyperbaric oxygen therapy’ and ‘stomatognathic diseases’ were used where applicable. Additional keywords like ‘radiation-induced damage’, ‘oral and facial diseases’, ‘medication-related osteonecrosis of the jaw’, and ‘oral and maxillofacial surgery’ were also included. Other terms were searched, specifically the names of other ischemic craniomaxillofacial diseases. The search was conducted from the inception of the databases to October 2024.

The titles and abstracts of the retrieved articles were initially reviewed, and full-text assessments were performed by two independent authors (He, C. and Huang, D.). Studies that were not published in English and that were unrelated to HBOT or ischemic craniomaxillofacial diseases were excluded. A complete review of the texts was carried out to ensure that the inclusion and exclusion criteria were met.

### 2.2. Inclusion and Exclusion Criteria

The inclusion criteria were as follows: (1) patients diagnosed with ischemic craniomaxillofacial diseases; (2) documentation of the use of HBOT as an adjunctive treatment; and (3) studies discussing the effects of HBOT on ischemia in craniomaxillofacial diseases. Given the relatively limited number of studies focusing on HBOT for these specific diseases, case reports and case series were also considered for review.

The exclusion criteria were as follows: (1) studies not published in English; and (2) studies unrelated to HBOT or craniomaxillofacial diseases.

## 3. Results and Discussion

From the database search, 131 articles were identified. Of the total literature, 68 were selected according to the selection criteria and objectives of the study.

### 3.1. History and Development of HBOT

The development of hyperbaric oxygen medicine has spanned several centuries. In 1662, British doctor Henshaw designed the first hyperbaric oxygen chamber, and he believed that hyperbaric oxygen was effective in treating diseases, which laid the foundation for HBOT [16]. In 1937, Behnke and Shaw pioneered the use of hyperbaric oxygen to treat decompression sickness, heralding the dawn of HBOT [17]. Two decades later, in 1960, Dutch scholar Boerema [18] published the groundbreaking book *Life Without Blood*. In it, he detailed experiments where pigs survived for 45 min inside a hyperbaric oxygen chamber with negligible blood cells, providing further evidence of the therapy’s remarkable potential therapeutic value. Since the 1970s, hyperbaric oxygen chambers have been increasingly utilized in medical settings worldwide [17]. With advancements in technology, HBOT is now employed for conditions such as carbon monoxide poisoning, hypoxic brain dysfunction, and central retinal artery obstruction, among others [19].

### 3.2. The Mechanism of HBOT for Ischemic Diseases in Craniomaxillofacial Surgery

The mechanisms by which HBOT cures diseases are currently a hot topic of research. The mechanisms underlying the therapeutic effects of HBOT involve the promotion of angiogenesis, antibacterial aspects, reduction in inflammation, and bone regeneration enhancement.

#### 3.2.1. The Effect of Promoting Angiogenesis

The primary challenge in managing ischemic craniofacial diseases is localized ischemia, which can significantly disrupt the normal structure and function of local tissues. This disruption often results in chronic refractory ischemic craniomaxillofacial diseases. Consequently, promoting tissue vascularization has emerged as a key area of research in the context of chronic non-healing diseases, with HBOT proving to be beneficial in enhancing revascularization.

The mechanisms by which hyperbaric oxygen promotes angiogenesis are related to the improvement of tissue hypoxia and the stimulation of growth factor secretion. Obviously, HBOT increases the concentration of oxygen in tissues, improving the hypoxic conditions and thereby enhancing cellular metabolism and function. Hypoxia-inducible factor-1 (HIF-1) is a key transcription factor, and its activation promotes the expression of various genes related to angiogenesis, such as vascular endothelial growth factor (VEGF). The study [20] by Pedro JC et al. indicates that the tissues after HBOT show an increase in the average number of HIF-1 positive cells, which subsequently induces an increase in VEGF-positive vascular structures. Furthermore, the elevated levels of HIF-1 stimulate the synthesis and release of VEGF, in turn promoting the proliferation, migration, and formation of new blood vessels by endothelial cells [21]. Simultaneously, many studies [22,23] have shown that HBOT effectively supports disease healing and optimizes the tissue microenvironment by promoting angiogenesis, restoring the blood supply to hypoxic tissues, enhancing cellular function and nutrition, generating collagen fibers, increasing elastic microfibril length, and lengthening telomeres in peripheral blood mononuclear cells.

HBOT has been shown to promote angiogenesis, as confirmed by multiple studies. The studies by Bhutani S and Vishwanath G [24] indicate that HBOT significantly increases the dissolved oxygen levels in the blood and accelerates angiogenesis, promoting collateral circulation and enhancing microcirculation in damaged areas to support new tissue growth. A clinical investigation [25] involving 20 participants demonstrated that HBOT significantly increased the density and area of blood and lymphatic vessels in the oral mucosa of individuals undergoing radiotherapy for craniomaxillofacial cancer.

HBOT enhances angiogenesis, further improving blood flow, oxygen transport capacity, and tissue nourishment. This has significant therapeutic potential for the treatment of ischemic diseases in the craniofacial region.

#### 3.2.2. The Effect of Antibacterial Effects

Due to the presence of a significant number of bacteria in the craniofacial region, ischemic craniofacial diseases often lead to chronic infections as a result of localized bacterial invasion and proliferation after tissue damage. This poses a considerable challenge in the treatment of such conditions. Fortunately, HBOT can effectively exhibit antimicrobial properties [7].

One of the reasons why HBOT directly kills or inhibits pathogenic bacteria is that it increases the partial pressure of oxygen in tissues, raising the levels of reactive oxygen species (ROS) within cells [4]. ROS are products of cellular metabolic processes, including oxide ions, peroxides, and oxygen radicals. ROS are significant in eliminating pathogens as they can directly affect the deoxyribonucleic acid, ribonucleic acid, proteins, and lipids of the pathogens, thereby killing bacteria or inhibiting their growth.

HBOT may have a synergistic effect when combined with certain antibiotics. This is primarily because hyperbaric oxygen enhances aerobic metabolism in bacteria, which, in turn, improves the efficacy of antibiotics that rely partially on this metabolic pathway, such as β-lactams, fluoroquinolones, and aminoglycosides [26]. In vitro and animal studies have shown that HBOT can enhance the efficacy of some antibiotics. Some scholars have emphasized its efficacy in treating infections caused by antibiotic-resistant pathogens. For instance, HBOT combined with tobramycin is more effective in treating *Staphylococcus aureus*-induced osteomyelitis than using tobramycin alone [27]. HBOT significantly increases the bactericidal effect of tobramycin exposure against *Pseudomonas aeruginosa* through counteracting the Pseudomonas aeruginosa biofilm micro-compartment phenomenon [28]. In an animal model of mediastinitis, the combination of vancomycin, teicoplanin, and linezolid with HBOT significantly improved the bactericidal effect against *Methicillin-resistant Staphylococcus aureus* [29].

As research continues to unveil its mechanisms and benefits, high-pressure oxygen therapy offers new hope for patients suffering from chronic infections, paving the way for more effective management strategies.

#### 3.2.3. The Effect of Anti-Inflammatory Responses

HBOT plays a crucial role in reducing inflammatory responses, a benefit that is likely attributed to the increased oxygen levels within the tissues. This elevated-oxygen environment meets the high metabolic demands of damaged tissues, thereby promoting healing and regeneration. Furthermore, the increased oxygen levels help to suppress the production and release of various inflammatory factors, thereby reducing the overall inflammatory response. Cytokines, such as Granulocyte Colony-Stimulating Factor (G-CSF), interleukin-6 (IL-6), interleukin-12p40 (IL-12p40), macrophage inflammatory protein-1β (MIP-1β), platelet-derived growth factor-BB (PDGF-BB), and interleukin-1 receptor antagonist (IL-1Ra), et cetera, play a crucial role in the inflammatory response. The levels of these factors are closely related to the severity of inflammation, typically showing a significant increase or decrease during infection or tissue injury. Recent studies have found that HBOT, as an adjunct treatment, may effectively improve the clinical symptoms of patients with necrotizing soft tissue infections. Its mechanism is believed to be associated with the reduction in G-CSF and IL-6 levels in plasma [30]. The decrease in these cytokines helps to suppress the inflammatory response, regulate the immune system, and potentially alleviate pathological conditions, thereby promoting tissue healing. IL-12p40, MIP-1β, and PDGF-BB play crucial roles in the inflammatory response within scar tissue; their reduction helps to alleviate local inflammation and thereby improves tissue healing. In contrast, IL-1Ra is an anti-inflammatory factor, and its increased expression helps to reduce tissue inflammation. According to a study [31] on the effects of HBOT on the expression of inflammatory factors in scar tissue, HBOT can effectively improve the inflammatory condition of scar tissue. Based on the results from H&E staining, immunohistochemical staining, and Western blot analysis, HBOT significantly reduces the levels of IL-12p40, MIP-1β, and PDGF-BB while increasing the levels of IL-1Ra. Therefore, this indicates that HBOT can enhance the microenvironment of scar tissue and provides a protective effect against the inflammatory responses during scar progression.

Neutrophils are one of the first cells recruited to the site of injury through an integrin-mediated recruitment process, and they play a crucial role in wound healing by releasing reactive oxygen species (ROS) and proteolytic enzymes. A reduction in the recruitment of neutrophils to the damaged area is beneficial for decreasing inflammation and promoting chronic wound healing. β2 integrin is one of the receptors that mediates the adhesion of neutrophils to endothelial cells. In experiments [32] involving HBOT for diabetic patients with chronic wounds, a significant decrease in the expression of β2 integrin on the surface of neutrophils was observed, and this lower expression level remained unchanged one month after the last treatment. This phenomenon indicates that HBOT not only promotes wound healing but also has good anti-inflammatory effects, which contributes to improving the healing process of chronic skin wounds.

By mitigating inflammation, HBOT contributes to a more favorable environment for tissue repair and recovery. This therapeutic approach can be particularly beneficial in managing chronic wounds, inflammatory conditions, and other scenarios where inflammation is a significant concern, ultimately leading to improved clinical outcomes and enhanced patient recovery.

#### 3.2.4. The Effect of Promoting Bone Regeneration

HBOT has shown promising results in promoting bone regeneration, an area of great interest in both clinical and research settings. The relationship between HBOT and bone regeneration is rooted in the therapy’s ability to enhance oxygen delivery to hypoxic or damaged tissues, which plays a critical role in the bone healing process. Researchers [32] conducted an in-depth analysis of the effects of HOBT on bone remodeling in a radiated rat fibula defect model. Rats with bone defects underwent HOBT for 0, 1, and 3 weeks, with sampling and analysis performed 6 weeks post-surgery. The micro-CT analysis showed that the new bone volume in the 3-week HOBT group was significantly increased, regardless of whether bone grafting was performed, highlighting the potential of HOBT in promoting bone tissue regeneration and repair. Moreover, a histological validation revealed that the percent of new bone area and the percent of blood vessel numbers were significantly elevated in the 1-week and 3-week HOBT groups, further confirming the positive impact of HOBT on bone remodeling.

Korucu et al. [33] conducted an in-depth study on the synergistic effects of HOBT and platelet-rich plasma (PRP) in the repair of cartilage injuries in rats. They randomly divided 32 rats into four groups: a control group, a PRP group, an HOBT group, and a combined HOBT and PRP group. After 30 days of different treatments, the researchers carefully observed the bone defect conditions in each group of rats. The results indicated that, in the group treated with the combination of HOBT and PRP, the volume of bone defects was significantly reduced, and there was noticeable formation of cartilage and new bone at the edges of the defects, demonstrating excellent treatment effects. Moreover, the area surrounding the defects gradually became smoother, indicating a healthier tissue structure. This finding suggests that the combination of these two therapies has a more significant advantage in promoting the regeneration of articular cartilage compared to using PRP or HOBT alone. Additionally, the study pointed out that, in rodent models, adjunctive HBOT could significantly accelerate the healing process of cartilage lesions caused by microfracture surgery. Thus, the combination of HOBT and PRP offers an effective new approach for the treatment of cartilage injuries and provides a valuable theoretical foundation for future clinical applications.

HBOT is also significant in promoting bone healing in diabetic patients. Research by Dias PC and colleagues suggests that HBOT enhances early bone regeneration in diabetic rats, highlighting its potential to mitigate the negative effects of diabetes on bone healing.

To sum up, these findings provide new experimental evidence for the application of HOBT in bone defect repair and indicate future research directions in related fields, particularly in enhancing bone regeneration efficiency and accelerating the rehabilitation processes, which hold substantial clinical application value.

### 3.3. Application of HBOT in the Treatment of Refractory Ischemic Craniomaxillofacial Diseases

HBOT for refractory ischemic craniomaxillofacial diseases has attracted much attention in recent years as an exploratory treatment approach. This section will delve into the clinical experience of HBOT in the field of craniomaxillofacial surgery, aiming to provide new ideas and directions for the application of HBOT in the treatment of these diseases.

#### 3.3.1. Medication-Related Osteonecrosis of the Jaw

Medication-related osteonecrosis of the jaw (MRONJ) is a serious pathological manifestation characterized by jaw necrosis resulting from the administration of specific medications, such as bisphosphonates and antiangiogenic agents, in the management of systemic disorders. According to the 2022 American Association of Oral and Maxillofacial Surgeons paper, the definition of MRONJ includes all the following elements: (1) current or previous treatment with antiresorptive therapy, alone or in combination with immune modulators or antiangiogenic medications, (2) exposed bone or bone that can be probed through an intraoral or extraoral fistula in the maxillofacial region, which persists for more than 8 weeks, and (3) no history of radiation therapy to the jaws or metastatic disease to the jaws [34]. This condition has emerged as a significant concern in the medical community over the past two decades. The widespread use of bisphosphonates and targeted tumor therapies has led to an escalation in the prominence of MRONJ, establishing it as a prevalent craniomaxillofacial disease. Estimates suggest that the prevalence of MRONJ among patients treated with bisphosphonates for osteoporosis or malignant tumors ranges between 0.001% and 0.2%. However, in cancer patients receiving intravenous zoledronate who undergo tooth extraction, the prevalence of MRONJ increases significantly, ranging from 1.6% to 14.8% [35].

Bisphosphonates and denosumab are medications used to treat conditions such as osteoporosis and bone cancer as they interfere with bone metabolism by inhibiting osteoclast activity. The mechanism by which bisphosphonates lead to jawbone necrosis is through the induction of osteoclast apoptosis via the inhibition of farnesyl pyrophosphate synthase. Denosumab and other antiangiogenic agents are bone resorption inhibitors that specifically target the receptor activator of the NF-kB ligand (RANKL) to inhibit osteoclast activity. Unfortunately, both bisphosphonates and antiangiogenic agents can also reduce the blood supply to the bone and disrupt normal bone metabolic processes, thereby affecting the repair and regeneration capacity of bone tissue [36]. Zolendronic acid inhibits and disrupts the proliferation, adhesion, and migration of human endothelial cells, decreases VEGF levels, and reduces angiogenesis, thereby suppressing tumor invasion and growth, while anti-angiogenic agents damage existing microvessels by increasing endothelial cell apoptosis. As these factors diminish the healing ability of the tissue, patients taking these medications are more prone to jaw osteonecrosis following localized surgical injuries.

The clinical features of drug-induced jaw necrosis commonly include pain, necrotic bone, gingival swelling, alveolar bone exposure, recurrent oral infections with purulent discharge, and the formation of extraoral fistulas. In extreme cases, pathological fractures may ensue, significantly impacting the patient’s quality of life and overall physical and mental well-being. HBOT has emerged as a potential adjunctive treatment for MRONJ (medication-related osteonecrosis of the jaw). In a randomized controlled study [37], 46 patients with drug-related bone necrosis were divided into two groups: a control group that received a standard surgical and antibiotic treatment and an experimental group that received the same standard treatment combined with HBOT. The results indicated that the experimental group showed symptom improvement in 39.7 weeks, whereas the control group took 67.9 weeks. This suggests that HBOT can effectively alleviate symptoms associated with drug-induced jaw necrosis, leading to pain reduction and better management of the affected tissue. Fatema CN [38] reported a case involving an 80-year-old patient with bisphosphonate-related jaw necrosis who underwent HBOT both before and after surgery. In our clinical practice, we have extensively used HBOT as an adjunct to surgery for MRONJ, and the results demonstrate that it significantly enhances wound healing and helps to prevent postoperative recurrence. The studies mentioned above highlighted the remarkable benefits of hyperbaric-oxygen-assisted treatment in controlling preoperative symptoms and facilitating postoperative healing for the patients.

#### 3.3.2. Craniomaxillofacial Pathologies After Radiotherapy

Radiotherapy is a treatment method that uses high-energy radiation (such as X-rays or particle beams) to kill or damage cancer cells, playing a crucial role in the treatment of certain specific malignancies and saving patients’ lives. However, while radiotherapy effectively destroys malignant tumors, it can also damage normal tissues, triggering a cascade of pathological processes such as osteonecrosis of the jaw and xerostomia (dry mouth) as complications. In fact, craniomaxillofacial pathologies, particularly osteoradionecrosis of the jaw (ORNJ), have undergone a rise in incidence in recent years and have become a common and difficult-to-treat ischemic condition in clinical practice.

Drawing from the existing literature, ORNJ is defined as a condition characterized by a non-healing, devitalized, and irradiated mandible, with or without associated soft tissue damage, that persists for at least 3 months [39]. ORNJ results from a combination of factors such as vascular damage, chronic inflammatory states in tissue, and bacterial infections. The ORNJ mechanism proposed by Marx RE is widely recognized; it points out that radiation-induced cellular damage leads to the formation of hypoxic, hypocellular, and avascular tissue, which can hinder healing and ultimately result in ORNJ [40]. Reduced angiogenesis plays a significant role in the pathogenesis of ORNJ. The study [41] by Dekker H and colleagues showed that, compared to the unirradiated control group, the number and density of blood vessels in the mandibles of irradiated patients were significantly reduced, indicating that radiation leads to vascular hypoplasia. Furthermore, the study also revealed that smaller vessels in the mandibular bone marrow were more susceptible to irradiation effects than larger vessels. After exposure to radiation doses exceeding 50 Gy, the average circumference and diameter of the vessels significantly increased, while the proportion of small vessels decreased significantly.

HBOT has gained widespread recognition among scholars as a valuable adjunct therapy in the management of radiation-related diseases since Mainous EG [42] pioneered the use of HBOT as a treatment for radioactive osteonecrosis and achieved promising outcomes. HBOT has shown significant effectiveness in certain cases, promoting wound healing and improving tissue hypoxia, although it cannot fully replace conventional medical measures such as surgical treatment and antibiotics yet [43,44]. Therefore, in medical practice, it is still essential to combine HBOT with other treatment methods based on the specific circumstances of the patient in order to achieve the best therapeutic outcomes.

HBOT can serve as a supplementary treatment alongside surgery for radiation-induced osteonecrosis due to its ability to stimulate blood vessel growth and expedite bone regeneration. A prospective study [45] led by Dang B et al. indicates that this therapy may lower the likelihood of developing jaw osteonecrosis after radiation treatment for head and neck tumors. However, there is some debate surrounding the use of HBOT for radiation-induced osteonecrosis. Sultan A [46] suggests considering HBOT as an additional option for high-risk patients who have not responded favorably to conservative measures and subsequent surgical intervention depending on the individual circumstances. Meanwhile, Forner LE [47] conducted a randomized clinical trial involving 114 patients, revealing that HBOT substantially reduces the occurrence of radioactive osteonecrosis following tooth extraction within the irradiated area. Similarly, Hampson NB’s [48] retrospective analysis of 43 patients with jaw radiation osteonecrosis revealed a remarkable 94% response rate to HBOT. In a survey conducted by Dieleman FJ et al. [49], the attitudes of oral and maxillofacial surgeons in the Netherlands towards the use of HBOT in the treatment of radioactive osteonecrosis were examined. The results indicated that these surgeons generally believed in the beneficial effects of HBOT for the management of radioactive osteonecrosis of the jaw. To achieve the best possible outcome, HBOT should be carried out simultaneously with surgical procedures and appropriate culture-directed antibiotic therapy [50,51].

Additionally, HBOT may contribute to the recovery of salivary glands in patients with head and neck cancer undergoing radiotherapy. It has demonstrated efficacy in alleviating dry mouth syndrome, prompting Hadley T et al. to propose its inclusion in the rehabilitation program for patients receiving head and neck radiotherapy [52]. Sherlock S et al. [53] conducted a randomized controlled study on 99 patients to assess the effectiveness of HBOT in treating radiation-induced dry mouth. The results indicated that HBOT effectively increases saliva production, thereby improving patients’ comfort and quality of life after treatment.

According to research by John G et al. [54], timely use of HBOT after radiation-induced optic neuropathy can effectively restore patients’ vision. Additionally, case reports [55,56] also demonstrate that timely use of HBOT may preserve vision in the eyes when they are less affected by radiation therapy.

HBOT significantly reduces the likelihood of pathological scar recurrence after radiotherapy while also enhancing patient satisfaction and improving their quality of life [57]. Furthermore, the reduction in scores on the Vancouver Scar Scale also confirms its effectiveness, providing a new and effective treatment approach for the post-radiation proliferation of pathological scars.

Taken together, these findings underscore the significant role of HBOT in both the prevention and treatment of craniomaxillofacial lesions after radiotherapy, offering hope to patients struggling with this debilitating condition.

#### 3.3.3. Chronic Hard-to-Heal Wounds in the Craniomaxillofacial Region

The healing of chronic wounds in the craniofacial region is influenced by various factors. Severe trauma, older age, and chronic infections can all impede the healing process. Underlying conditions such as diabetes and atherosclerosis further exacerbate these challenges. Therefore, the application of HBOT to improve the prognosis of chronic wounds is crucial for the treatment of non-healing injuries in the craniofacial region.

Jaw osteomyelitis is a prevalent inflammatory condition affecting the jaw, primarily caused by bacterial infections and physical or chemical factors. Among these, chronic infectious osteomyelitis stands out as the most common type. This condition is typically triggered by dental issues such as periodontitis, pulpitis, apical inflammation, or pericoronitis associated with wisdom teeth. A systematic literature review [58] of 45 studies involving 460 patients with chronic osteomyelitis found that adjunctive HBOT significantly improved the condition, showing effectiveness in 80% of the cohort studies and 95% of the case studies, thus supporting its beneficial role in refractory cases.

Craniomaxillofacial trauma, commonly resulting from falls, car accidents, and altercations, often entails soft tissue damage, dental injuries, and fractures. When the skin and mucosal surfaces sustain extensive injuries, they are prone to prolonged healing or secondary infections. Furthermore, severe maxillofacial fractures can exhibit poor healing or become infected. HBOT is beneficial in promoting the healing of poorly healing fractures and jawbone recovery [59,60].

Additionally, this therapeutic modality can promote periodontal healing and improve tooth survival rates in patients with dental trauma [61]. In a study [62] involving 69 participants and 138 teeth, the researchers compared the impact of HBOT therapy on the prognosis of replantation. The results indicated that the hyperbaric oxygen group showed significant improvements in periodontal healing, tooth survival rates, and pulp healing compared to the control group, which holds important clinical significance.

Hard-to-heal wound healing in the craniomaxillofacial region is also prevalent among cancer patients undergoing diseased tissue removal and those with systemic conditions like diabetes or malnutrition. For these patients, HBOT can effectively promote chronic wound healing and enhance patient outcomes.

Jiang W et al. [63] conducted a randomized controlled trial involving 83 patients with hard-to-heal wound healing after pharyngeal cancer surgery. Their aim was to investigate the efficacy of HBOT in facilitating advanced wound healing post-surgery. Their findings revealed that HBOT stimulated the growth of fresh granulation tissue and reduced the wound healing time following necrotic tissue excision, pharyngeal fistula repair, and infection debridement.

HBOT has shown significant advantages in improving healing outcomes for patients with hard-to-heal wounds. Studies [64] indicate that patients who undergo hyperbaric oxygen treatment experience markedly better wound healing results compared to those who do not receive treatment, evidenced by a faster reduction in ulcer size, shorter healing time, and a significant increase in transcutaneous oxygen measurements.

Moreover, HBOT has demonstrated significant medical value in scar reduction due to promoting angiogenesis and reducing the inflammatory response., particularly playing a positive role in the comprehensive surgical treatment of craniomaxillofacial burns. In the comprehensive surgical management of craniomaxillofacial burns, adjunctive HBOT can mitigate bacterial proliferation and facilitate hard-to-heal wound healing [65,66].

#### 3.3.4. Promotion of Graft and Flap Regeneration

Facial and neck tissues are often compromised by severe trauma or tumor treatment, necessitating the restoration or transplantation of healthy tissue. Skin flap transplantation is a crucial technique for addressing facial deformities or defects. However, this procedure frequently encounters challenges, such as blood supply obstacles and difficulties in flap survival. HBOT emerges as a valuable adjunctive measure to salvage compromised flaps and grafts. By mitigating hypoxia-induced damage, enhancing fibroblast function and collagen synthesis, stimulating angiogenesis, and suppressing ischemia-reperfusion injury, it can significantly improve the survival rate of flaps and grafts [67].

A study has reported a significant increase in the survival rate of implants following prophylactic HBOT [68]. In another study involving nine children with facial defects, Camison L’s study [69] observed that the immediate application of HBOT following large cartilage skin grafts enhanced flap survival. Furthermore, HBOT holds promise in plastic surgery. Tambasco D et al. [70] posit that this therapy is useful in reconstructive plastic surgery because it can alleviate early postoperative ecchymosis and edema, improve scar formation, reduce inflammation, and expedite healing. Notably, hyperbaric oxygen emerges as an efficacious treatment option for wound dehiscence after cleft palate repair. Case reports [71] indicate that early HBOT in cases of wound dehiscence or perforation (palatal fistula) following cleft palate repair can facilitate fistula narrowing and promote granulation tissue growth at the wound margin.

HBOT has shown potential clinical effects as an adjunctive treatment for hair transplant surgery. In a study [72], 34 patients with hair loss were randomly divided into a control group and an HBOT group. The control group received a conventional surgical treatment, while the HBOT group received additional HBOT. The results indicate that the HBOT group experienced significantly reduced postoperative itching and folliculitis and had a lower rate of hair loss compared to the control group. However, there was no significant difference in the hair survival rates between the HBOT and control groups at 9 months post-surgery. This suggests that HBOT can effectively protect transplanted hair follicles in the short term after hair transplantation, providing strong evidence for its use as an adjunctive therapy in hair transplant procedures.

**Figure 1:**
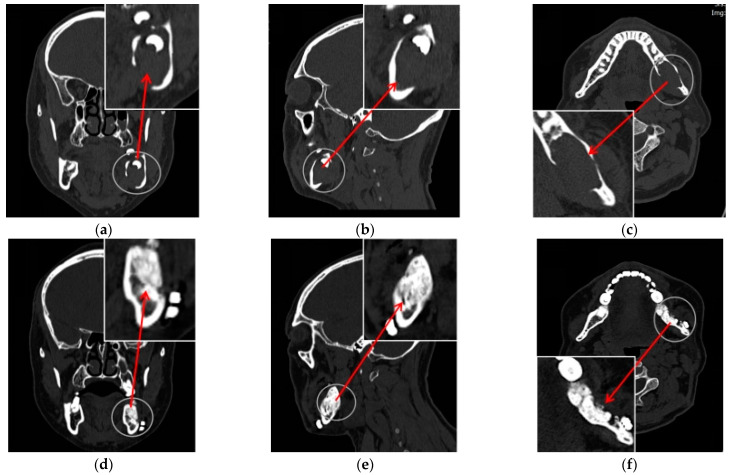
A case of jaw cysts treated with HBOT as adjunctive therapy. (**a**–**c**) Show the preoperative computed tomography (CT) images of the patient’s cysts; (**d**–**f**) show the six-month postoperative CT images after surgery.

Cysts are a common occurrence in the oral maxillofacial region. The curettage of a jaw cyst can effectively remove the diseased tissue, but the remaining cavity may affect the morphology of the jaw, decrease its strength, and increase the risk of pathological fractures. Bone grafting is often employed to reconstruct the jaw cavity, reinforce the bone, and prevent pathological fractures; however, the effectiveness of bone regeneration may not be optimal due to large bone defects and factors such as infection at the lesion site. HBOT plays a significant role in enhancing the effectiveness of bone grafting. Our team [73] undertook a randomized controlled study involving 90 patients with jaw cysts to investigate the impact of HBOT as an adjunctive treatment on the prognosis of jaw cyst removal. The results indicated that HBOT reduced postoperative infections and improved the rate of skeletal healing (Figure 1).

In summary, HBOT offers presents a promising new approach for tissue defect repair, and its potential benefits deserve further investigation and discussion.

#### 3.3.5. Neurological Diseases

Facial muscle movement is primarily controlled by the facial nerve, and dysfunction of this nerve frequently results in facial paralysis. Due to the lengthy course of the facial nerve within the narrow bony canal and the lack of local blood supply, facial nerve lesions are believed to be related to ischemia and swelling. Although the quality of evidence from studies is limited, corticosteroid-assisted HBOT has been found to shorten the recovery time of facial paralysis and facilitate complete recovery [74]. Moreover, the study [75] conducted by Brenna CT and colleagues found that HBOT is a promising intervention that can treat peripheral nerve injuries caused by tissue ischemia during the perioperative period.

HBOT has demonstrated efficacy in treating chronic secondary neuropathic pain conditions, including plexus neuropathy, postherpetic nerve pain, and trigeminal neuralgia resulting from radiation and chemotherapy. Clinical trials involving HBOT [76] (administered at 1.8 ATA for 70 min once daily for 10 days) have shown it to be a safe and effective treatment for relieving trigeminal neuralgia. The analgesic effects of this therapy can persist for at least 60 days, during which time there is a significant reduction in the required dose of carbamazepine.

HBOT has been found to be effective for idiopathic sudden sensorineural hearing loss, particularly in restoring low-frequency hearing and improving severe to profound initial hearing levels [77]. Recent studies have further highlighted the significant benefits of HBOT in enhancing functional recovery after optic nerve and auditory nerve disorders, offering new hope for the rehabilitation of craniomaxillofacial nerve diseases. Case reports [78,79] also indicate that immediate HBOT is a worthwhile treatment option for ischemic and mechanical injuries to the optic nerve following trauma as it promotes angiogenesis, thereby aiding in nerve recovery.

#### 3.3.6. Others

Regarding skin health, HBOT has demonstrated positive outcomes in enhancing facial skin conditions. Nishizaka T [80] conducted a study involving seven healthy female participants with facial age spots. After 12 weeks of HBOT, the size of the age spots reduced to 82% of their original size. This finding indicates that hyperbaric oxygen can boost oxidative metabolism and diminish the appearance of age spots. In another randomized controlled trial led by Wiser I et al. [81], eight cosmetic patients undergoing chemical peeling received HBOT. The results showed [82] that this treatment accelerated wound healing post-chemical peeling and minimized local side effects.

### 3.4. The Side Effects of HBOT

HBOT is widely recognized for its applications in the treatment of various medical conditions, but its side effects also deserve our attention. The adverse reactions of HBOT include ear discomfort, sinus and paranasal sinus barotrauma, hypoglycemia, oxygen-induced seizures, and claustrophobia, which may be caused by the adverse effects of pressure as well as the adverse effects of oxygen [83]. The most frequent side effect of HBOT is ear discomfort [82]. During the HBOT treatment process, patients may experience changes in ear pressure, leading to ear pain or temporary hearing loss, a condition known as barotrauma. Similar to the ear, the pressure changes during HBOT can also affect the sinuses, causing sinus pain or discomfort, particularly if patients struggle to equalize the pressure in their sinuses during treatment. Fortunately, these symptoms are self-limiting and can be easily and effectively alleviated through patient coaching and topical medications. Diabetic patients [83] should closely monitor their blood glucose levels before and after HBOT as there is a risk of hypoglycemia during the treatment. In the past, some patients undergoing HBOT might have experienced symptoms of oxygen toxicity, such as headaches, nausea, and dizziness, and, in severe cases, could have developed seizures or altered consciousness; prolonged exposure to hyperbaric oxygen could also lead to lesions like alveolar rupture or pulmonary edema. Claustrophobia, caused by the confined space within the treatment chamber, is a contraindication for HBOT. To prevent such adverse reactions, doctors must carefully select patients with no history of claustrophobia for treatment. With advancements in modern hyperbaric oxygen equipment and deeper research into HBOT, the treatment has become safer and more reliable. Based on the findings from A Retrospective Analysis of Adverse Events in HBOT (2012–2015) [84], the incidence of adverse events was 0.68% in 1.5 million treatments analyzed, and medically severe events were extremely uncommon, with fewer than 0.05 instances of oxygen toxicity per 1000 treatments and only a single confirmed case of pneumothorax. In conclusion, HBOT is safe. By ensuring careful adherence to these indications, healthcare providers can maximize the benefits of HBOT while minimizing the risks, ultimately enhancing patient outcomes.

It is crucial to emphasize that, for most pathologies, HBOT serves as a complementary component within a broader treatment approach, alongside other modalities, particularly surgery. Prior to initiating HBOT, clinicians must assess the patient’s overall condition, determine its appropriateness, and devise a tailored treatment plan.

### 3.5. Prospects and Future

Despite significant advancements in the application of HBOT as an adjunctive treatment for refractory ischemic craniomaxillofacial diseases, numerous research gaps still need to be addressed. These encompass the following: (1) the conduct of large-scale randomized controlled trials to elucidate the efficacy and specific indications of HBOT; (2) the exploration of the standardized parameters and treatment protocols tailored for various craniomaxillofacial pathologies; (3) the establishment of clinical guidelines to standardize the indications and treatment plans for HBOT; and (4) the elucidation of the precise molecular mechanisms underlying HBOT’s effects at the molecular and cellular levels. As the research in these areas progresses, HBOT is poised to become an indispensable component of the therapeutic arsenal for a wide range of refractory ischemic craniomaxillofacial diseases, thereby contributing to the ongoing improvement in treatment outcomes for related pathologies.

### 3.6. Limitations

Despite many studies indicating that HBOT demonstrates significant therapeutic effects in the treatment of refractory ischemic craniomaxillofacial diseases, this review does have several limitations. First, due to the scope and nature of this review, we may have overlooked some relevant studies, particularly those published in non-English languages or in journals not indexed in the databases we searched. This may have introduced bias into our results and conclusions. Second, the quality and methodological rigor of the studies included in our review varied, which may have influenced the reliability and validity of our findings. To mitigate this, we critically appraised each study and considered the potential impact of their methodological limitations in our analysis. We recognize these limitations and suggest that future studies address these gaps to provide a more comprehensive understanding of the role of HBOT in the management of craniomaxillofacial diseases.

## 4. Conclusions

Both extensive research and clinical practice have demonstrated that HBOT serves as a promising adjunctive treatment, exerting a positive influence on refractory ischemic craniomaxillofacial diseases. HBOT provides a valuable supplement to the existing treatment options, especially in cases where traditional surgical interventions alone are inadequate, such as osteonecrosis, chronic infections, and non-healing wounds in the craniomaxillofacial region. Owing to its multifaceted therapeutic mechanisms, which encompass the promotion of angiogenesis, antibacterial effects, inflammation reduction, and enhancement of bone regeneration, HBOT has the potential to significantly improve the clinical outcomes and enhance the quality of life for those patients suffering from these conditions. As our comprehension of its underlying mechanisms continues to deepen, HBOT is poised to play an increasingly pivotal role in the therapeutic landscape, reshaping the management of refractory ischemic craniomaxillofacial diseases and driving advancements in healthcare solutions.

## Data Availability

Not applicable.
